# Microencapsulation Efficiency of Carboxymethylcellulose, Gelatin, Maltodextrin, and Acacia for Aroma Preservation in Jasmine Instant Tea

**DOI:** 10.3390/gels10100670

**Published:** 2024-10-21

**Authors:** Muneeba Naseer Chaudhary, Xiaolin Li, Siyue Yang, Damao Wang, Liyong Luo, Liang Zeng, Wei Luo

**Affiliations:** 1 Integrative Science Center of Germplasm Creation in Western China (CHONGQING) Science City, College of Food Science, Southwest University, Chongqing 400715, China; muneeba.ch22@outlook.com (M.N.C.); lixiaolin0804@163.com (X.L.); yangsiyue044@163.com (S.Y.); liyongluo1979@126.com (L.L.); 2 College of Food Science, Southwest University, Chongqing 400715, China; wangdamao@swu.edu.cn; 3 Chongqing Key Laboratory of Speciality Food Co-Built by Sichuan and Chongqing, Southwest University, Chongqing 400715, China

**Keywords:** microencapsulation, instant tea, aroma preservation, spray drying, encapsulating agents, volatile organic compounds

## Abstract

Enhancing the sensory appeal of jasmine instant tea, particularly its aroma, poses a significant challenge due to the loss of volatile organic compounds during conventional processing. This study introduces a novel approach to address this issue through the application of microencapsulation techniques, aimed at preserving these key aromatic elements. Our investigation focused on the encapsulating agents gelatin, acacia gum, carboxymethylcellulose (CMC), and maltodextrin, chosen for their compatibility with the volatile organic compounds of tea. A statistical analysis was conducted on the analytical results through comprehensive analytical techniques like Principal Component Analysis (PCA), Orthogonal Partial Least Squares Discriminant Analysis (OPLS-DA), and Variable Importance in Projection (VIP) analysis for microcapsule characterization. The statistical analysis revealed gelatin to be a particularly effective encapsulating medium, preserving an aroma profile more akin to fresh tea. The statistical analysis confirmed the reliability of these findings, highlighting the potential of microencapsulation in refining the quality of jasmine instant tea products. The results of this research suggest that microencapsulation could be instrumental in improving the sensory quality and shelf life of instant tea products, offering new opportunities for product enhancement in the beverage industry.

## 1. Introduction

The development of jasmine instant tea, noted for its convenience, presents a distinct set of challenges within food science, particularly in preserving its sensory qualities and shelf life [1,2]. Central to this challenge is the retention of volatile organic compounds, which are fundamental in shaping consumer preferences and determining the overall quality of the product [3]. The standard processing of jasmine instant tea, which includes extraction, concentration, drying, and often granulation [4], typically results in a notable loss of aromatic compounds [5], thereby diminishing the tea’s sensory appeal and perceived freshness. Addressing this issue, the current study investigates the application of microencapsulation technology as a novel approach to preserving aroma [6], aiming to surmount the existing production limitations while maintaining an equilibrium between processing efficacy and product quality.

Preserving aroma in jasmine instant tea involves a critical balance between protecting volatile organic compounds and optimizing the encapsulation process [7]. These compounds are highly susceptible to environmental factors such as oxygen [8], light, and temperature [9], prevalent during processing. This complexity is enhanced due to the diversity of the aromatic compounds, each responding uniquely to environmental conditions, necessitating a customized encapsulation approach. The key challenge lies in identifying and refining microencapsulation techniques to maximize aroma retention without compromising the tea’s natural quality and flavor [10]. Microencapsulation works by surrounding volatile organic compounds with a protective coating, forming a barrier that shields these compounds from degradation during processing and storage. By enclosing these compounds within a protective matrix, microencapsulation minimizes their exposure to environmental factors like heat, moisture, and oxygen, which can otherwise result in aroma loss or unwanted chemical reactions [11,12]. This technique ensures that these aroma compounds are only released when the tea is brewed, preserving both its flavor and natural integrity [13]. This helps maintain the sensory qualities of the tea. The sensory qualities of a product play a crucial role as they significantly impact a consumer’s decision to make an initial purchase [14]. To maintain the natural quality of tea, such as retaining antioxidants, polyphenols, and catechins, it is crucial to carefully control processing temperatures and use encapsulation materials that are safe for food and do not react with the tea’s natural compounds [15].

Using encapsulation materials like gelatin, maltodextrin, and gum arabic can effectively preserve both aroma and bioactive compounds without altering the tea’s natural flavor profile [15]. Recent studies have also highlighted the importance of preserving the bioactive components of jasmine instant tea to retain its health benefits and sensory qualities. Polyphenols, including catechins and flavonoids, are particularly valued for their potent antioxidant properties and their ability to reduce oxidative stress and inflammation [16]. The use of various types of microcapsules can modify a product’s texture, aroma, appearance, and taste. Microencapsulation offers advantages such as easier handling and better control over the release and solubilization of active ingredients, creating substantial opportunities for innovation in food science and processing [17].

Recent advancements in instant tea processing have focused on microencapsulation to enhance aroma profiles. To achieve successful encapsulation, a range of coating materials are used, each with unique functional properties. Commonly used materials include carboxymethylcellulose (CMC), gelatin, maltodextrin, and acacia gum, all known for their excellent film-forming abilities, biocompatibility, and protective qualities for sensitive ingredients [6,12]. Carboxymethylcellulose (CMC) is a cellulose derivative valued for its capacity to stabilize emulsions and create strong, flexible films around encapsulated particles. Recent studies, like those by Yildirim-Yalcin et al. [18], have shown CMC’s effectiveness in enhancing the thermal stability of encapsulated flavors, thereby extending shelf life. Gelatin is widely used in microencapsulation due to its unique gelling properties and its ability to form durable capsules that protect volatile organic compounds from oxidation. According to Luo, Hossen [19], gelatin-based microcapsules have been effective in retaining aromatic compounds across various food matrices, ensuring flavor preservation during storage. Maltodextrin is another popular encapsulating agent, known for its low viscosity and high solubility, making it ideal for spray-drying applications. Research by Li, Pan [20] indicates that maltodextrin effectively forms stable microcapsules that maintain the integrity of sensitive flavors and fragrances under diverse environmental conditions. Acacia gum, or gum arabic, is recognized for its emulsifying properties and its ability to form stable films that safeguard encapsulated ingredients. Recent findings by Mazumder and Ranganathan [21] suggest that acacia gum enhances the encapsulation efficiency and controlled release of flavors, making it particularly suitable for encapsulating volatile organic compounds in jasmine instant tea and other beverages.

In this study, to assess the effectiveness of jasmine instant tea microcapsules in protecting aromatic compounds, several analytical techniques were used for chemical characterization. Spectrophotometric methods were used to determine the encapsulation efficiency of the microcapsules by measuring total phenolic content and antioxidant activity. The total phenolic content (TPC) was determined using the Folin–Ciocalteu method, which measures phenolic compounds, which are important for maintaining the health benefits and quality of the tea. High levels of phenolics indicate better preservation of the tea’s bioactive compounds within the microcapsules. Antioxidant activity was measured by a DPPH (2,2-diphenyl-1-picrylhydrazyl) assay [22]. Gas Chromatography–Mass Spectrometry (GC-MS) was used to identify and quantify volatile organic compounds responsible for the jasmine aroma. This technique allows for precise detection of VOCs, which are crucial to evaluate the effectiveness of the microencapsulation in retaining these sensitive aromatics [23].

Physical characterization of microcapsules is essential for understanding their structural properties, which directly influence their performance in protecting the encapsulated compounds, such as flavors, antioxidants, and other bioactives. For instant tea microcapsules, physical properties like size, shape, surface morphology, and moisture content affect factors such as solubility, stability, and the release profile of aromatic compounds [24]. Particle Size Analysis measures the size distribution of microcapsules, helping us to assess the uniformity and potential solubility behavior of the microcapsules. This affects the dissolution rate and bioavailability. Smaller, uniform microcapsules often dissolve more quickly and uniformly, improving the rehydration properties [25]. Optimizing particle size distribution can greatly enhance the taste, texture, and rehydration effectiveness of instant tea powders [26].

Morphological features, like surface smoothness and porosity, are crucial in shaping the physical properties of jasmine instant tea powders. Scanning electron microscopy (SEM) is used to observe the surface structure, shape, and morphology of the microcapsules. It provides high-resolution images that reveal surface smoothness, cracks, and pores, essential for evaluating protective capabilities. Smooth surfaces and low porosity help improve the flowability and reduce moisture absorption, which protects volatile organic compounds like jasmine tea aromas from degradation [27]. Moisture content measurements evaluate the water retention properties of the microcapsules, which are crucial for protecting sensitive compounds like volatile organic compounds. High moisture content in micro-capsules can impact stability during storage, potentially causing shifts in the wall materials from a glassy state to an amorphous rubbery state. This shift may lead to degradation and subsequent release of the core material [28].

This research focuses on optimizing microencapsulation methods tailored to jasmine instant tea, addressing the challenge of preserving aroma during the manufacturing process. The goal is to develop a product that retains the sensory qualities of freshly brewed tea, as traditional processing often causes significant aroma loss due to high temperatures and air exposure. This study contributes novel insights on the microencapsulation of tea aromas

## 2. Results and Discussion

### 2.1. Microencapsulation Efficiency

**Figure 1:**
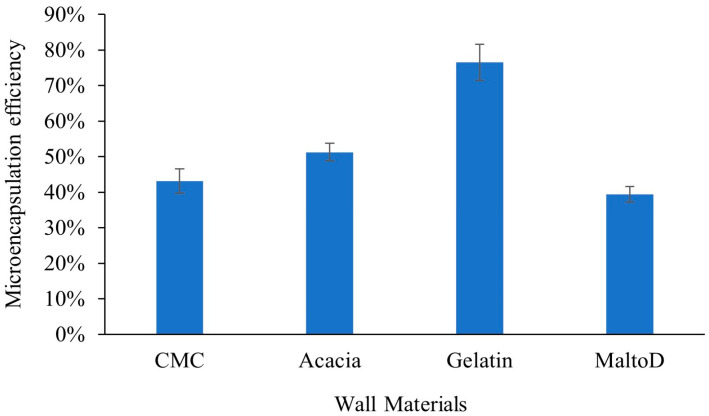
Encapsulation efficiency of various wall materials. Figure illustrates encapsulation efficiency observed for different wall materials, including gelatin, CMC, acacia gum, and maltodextrin. Data points are presented with error bars indicating standard deviations. Each measurement was replicated three times. CMC—carboxymethylcellulose.

Spectrophotometric methods were used to determine the encapsulation efficiency of the microcapsules, allowing us to measure how well the core material (aromatic compounds, phenolics) was encapsulated. The amounts of phenolic or other bioactive compounds released from the microcapsules were measured before and after encapsulation. High encapsulation efficiency ensures that the bioactive compounds remain protected during processing and storage, maintaining the quality and aroma of the tea. Figure 1 presents the encapsulation efficiency of four different wall materials, evaluated based on total polyphenol content. The extract obtained contained 192.26 mg/g of polyphenol (calculated based on catechins) per gram of dry extract. The polyphenol contents in the encapsulated powders are shown in Figure 1. Gelatin encapsulation demonstrated higher efficiency, ranging from 74% to 79%, while maltodextrin had the lowest, ranging from 37% to 41%. This difference can be attributed to the structure of different wall materials. This finding is in accordance with other studies [29,30]. Gelatin, as a protein with various functional groups, such as amino, carboxyl, and hydroxyl groups, can form hydrogen bonds and hydrophobic interactions with the hydroxyl and other groups in polyphenol molecules, enabling more effective encapsulation and stabilization of polyphenols. Additionally, the high molecular weight and multifunctional groups of gelatin enable it to provide greater efficiency and stability during the encapsulation process [30].

### 2.2. Polyphenol Content, Flavonoid Content, and Antioxidant Capacity

**Figure 2:**
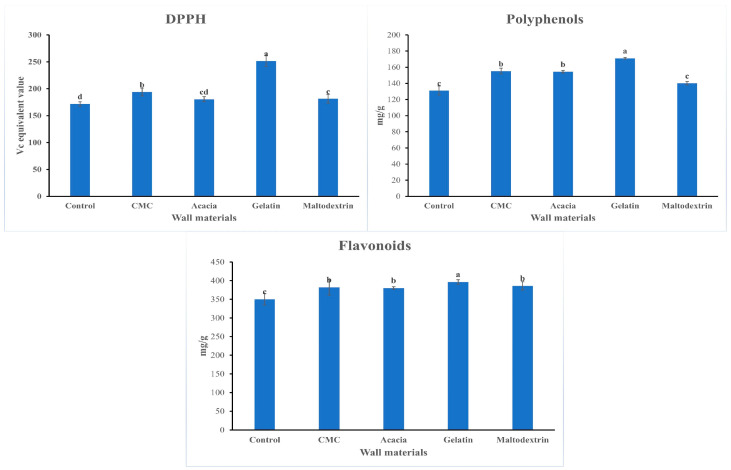
Effects of different encapsulation agents on antioxidant, polyphenol, and flavonoid contents of jasmine instant tea (CMC—carboxymethylcellulose). Different letters indicate statistically different at *p* < 0.05.

Polyphenols are known to be heat-sensitive compounds [31], which makes them prone to degradation during the concentration and drying processes. The prolonged exposure to high temperatures during these stages, along with the introduction of oxygen during spray drying, likely contributes to the significant reduction in polyphenol content [32]. This oxidative environment can accelerate the breakdown of polyphenols, leading to a loss of their antioxidant properties. Figure 2 highlights that, despite these challenges, the microencapsulation process effectively preserved the polyphenol content, flavonoid levels, and antioxidant capacity in the jasmine instant tea. This is consistent with previous studies [33,34], which suggest that microencapsulation is a reliable technique for safeguarding the bioactive compounds and functional properties of tea during processing. The encapsulation process helps protect these sensitive compounds from heat and oxygen exposure, thereby enhancing the stability and retention of key antioxidants.

Among the encapsulating agents evaluated, gelatin-based microcapsules showed the highest polyphenol content and antioxidant activity in the final product. Gelatin’s superior performance can be attributed to its excellent properties as a microencapsulation material. It forms a stable, flexible matrix that effectively protects polyphenols from oxidative degradation and environmental stress during both processing and storage [35]. As a result, polyphenols encapsulated in gelatin remain more stable and bioavailable, which translates into enhanced antioxidant activity in the final product. In contrast, the maltodextrin-based microcapsules exhibited the lowest polyphenol content and antioxidant properties. This suggests that, compared to gelatin, maltodextrin may be less effective in protecting and retaining these bioactive compounds during the encapsulation process. The differing performances of the encapsulating agents underscore the importance of selecting appropriate wall materials to optimize the retention of polyphenols and the overall antioxidant capacity of jasmine instant tea.

### 2.3. Physical Characterization

The particle size distribution of the microencapsulated compounds is a crucial factor determining the release rate and sensory impact of the encapsulated samples.

Figure S1 in the Supplementary Materials presents the particle size metrics of different wall materials. The median particle sizes (Dx (90)) of the control, CMC, acacia, gelatin, and maltodextrin are 12.71 ± 0.10 μm, 131.67 ± 0.67 μm, 10.71 ± 0.11 μm, 126.58 ± 5.65 μm, and 14.27 ± 0.17 μm. The control group shows a concentrated particle size distribution between 1.36 µm and 81.01 µm. Maltodextrin-based microcapsules exhibit a similar but slightly narrower distribution, indicating smaller and more uniform particles. In contrast, CMC-based microcapsules display a much broader size distribution, with a peak around 13.56 µm and a range extending up to 374.85 µm, suggesting wide variance in particle size. Gelatin-based microcapsules are smaller than CMC, with a peak near 7.65 µm and extending up to 374.85 µm. Acacia-based microcapsules have a distribution similar to the control and maltodextrin groups. These findings highlight that the choice of encapsulation material significantly affects the particle size distribution of microcapsules, which, in turn, may influence their functional properties and potential applications.

The particle size of microcapsules is typically correlated with their release characteristics. Smaller microcapsules ranging from 1 to 10 μm, due to their relatively larger surface area, generally exhibit faster release rates, whereas larger microcapsules ranging from 100 to 500 μm may facilitate a slower and more sustained release. However, smaller microcapsules may have higher solubility, which means they have better application prospects for instant tea [25]. In this study, the microcapsules prepared with gelatin and CMC have a larger particle size, which may indicate better retention of fragrance and nutrients. However, this could also result in poorer solubility. In contrast, microcapsules made with maltodextrin and acacia have smaller particle sizes, so they might not retain the fragrance as effectively [25].

Maintaining low moisture content is essential for preserving the aroma, stability, and quality of instant tea powder during storage and handling. In this study, the moisture contents of the gelatin, CMC, acacia, and maltodextrin were recorded to be 3.23%, 3.98%, 4.67%, and 4.98%, respectively, demonstrating the superior moisture retention of gelatin. Meanwhile, the moisture content of the control sample was higher, at 6.02%. For enhanced stability, it is recommended that the moisture content for jasmine instant tea powder be maintained between 3% and 5% [36].

**Figure 3:**
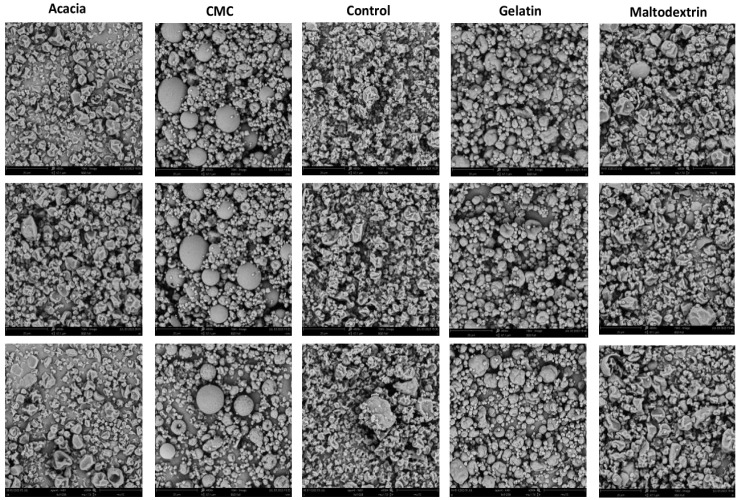
Scanning Electron Microscopy (SEM) images of microencapsulations produced with various wall materials. The images show the morphology and surface characteristics of microencapsulations made from gelatin, acacia gum, carboxymethylcellulose (CMC), and maltodextrin. Each image highlights the structural differences and surface textures of the microencapsulations depending on the wall material used.

The Scanning Electron Microscopy (SEM) image (Figure 3) confirms the particle size distribution of different wall materials. The particle sizes of the control group, gum arabic, and maltodextrin are smaller and more uniform, whereas the particle sizes of gelatin and CMC are larger and more variable.

### 2.4. Analysis of Aroma

The volatile organic compounds analyzed by SPME-GC-MS are presented in the Supplementary Materials (Table S1), and a chromatogram of each sample is exhibited Figure S2 in the Supplementary Materials. The chemometric analysis, via Principal Component Analysis (PCA), was instrumental in distinguishing the aroma profiles of the microencapsulated jasmine instant tea. The PCA plots provide a visual and quantitative assessment of the distribution and variance of the samples, indicating the relative success of each encapsulating agent in preserving the aromatic integrity of the jasmine instant tea.

**Figure 4:**
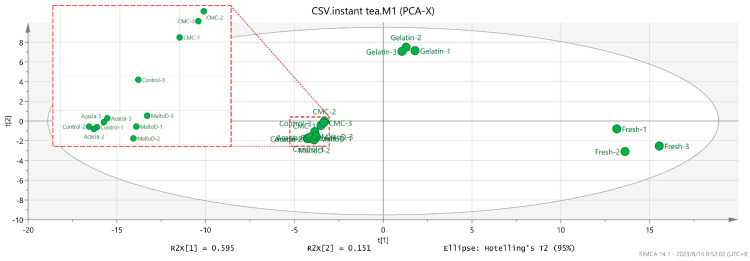
Principal Component Analysis (PCA) of microencapsulations produced from different wall materials compared to a sample made from fresh tea. The PCA plot shows the distribution and clustering of microencapsulated samples (gelatin, acacia gum, carboxymethylcellulose (CMC), maltodextrin) and fresh tea based on their aroma profiles.

As shown in Figure 4 the PCA (Principal Component Analysis) groups the analyzed samples into different clusters based on their volatile organic compound profiles. The variance explained by the first principal component (PC1) is 59.5%, and by the second principal component (PC2) is 15.1%. Through this analysis, it was found that tea brewed from tea leaves formed a distinct cluster compared to the samples prepared from jasmine instant tea. This clustering highlights the significant difference in the volatile profiles, likely due to the concentration and drying processes involved in the production of instant tea [37]. The high temperatures and vacuum conditions typically used during concentration and spray drying can cause the evaporation or degradation of volatile organic compounds, leading to a reduced aromatic profile in instant tea [38].

The PCA results also allowed the differentiation of jasmine instant tea samples based on the encapsulating agents used. For instance, samples encapsulated with gelatin showed closer clustering to the fresh tea reference point compared to other encapsulating agents like acacia gum, carboxymethylcellulose (CMC), and maltodextrin. This indicates that gelatin was more effective in preserving volatile organic compounds that contribute to the aroma of the tea. The ability of gelatin to retain these key aromatic compounds could be attributed to its molecular structure, which forms a dense, protective matrix around the volatile organic compounds, limiting their diffusion and evaporation during the spray-drying process [39].

Furthermore, the encapsulation efficiency of gelatin is likely influenced by the size of the microcapsules, as seen in Supplementary Materials Figures S1 and S3. The larger size of the gelatin-based microcapsules allows for greater encapsulation capacity and slower release rates, resulting in better retention of the volatile organic compounds. This suggests a potential correlation between the microcapsule size, the release dynamics of the aromatic compounds, and the overall sensory quality of the tea.

Our PCA findings (Figure 4) support the conclusion that different encapsulating agents influence aroma retention in jasmine instant tea to varying degrees. Specifically, the success of gelatin in preserving key volatile organic compounds suggests it may be an optimal choice for encapsulation in instant tea products that prioritize high sensory quality.

### 2.5. Orthogonal Partial Least Squares Discriminant Analysis (OPLS-DA)

#### 2.5.1. Permuted Value

An OPLS-DA model was used to further identify which specific aromatic components contribute to the differences between microcapsules prepared with various wall materials. The permutation test for the jasmine instant tea (Figure S3 in the Supplementary Materials) demonstrated that the model possesses predictive relevance. The R2 and Q2 values for the original model (R2 = 0.463, Q2 = −0.971) stand out when compared to the distribution of the permuted values, with intercepts of R2 = (0.0, 0.463) and Q2 = (0.0, −0.971). The non-interception of the zero line by the permuted R2 and Q2 values reinforces the model’s robustness against overfitting. The permutation tests confirm that the OPLS-DA models captured not merely noise, but rather, genuine underlying patterns in the data related to the microencapsulation’s effect on aroma preservation. The clear separation between the permuted and original model statistics indicates that the encapsulation process significantly impacts aroma profile retention, with certain treatments demonstrating a stronger preservation effect, as previously observed in the PCA and OPLS-DA results.

#### 2.5.2. OPLS-DA Plots

The OPLS-DA method was applied to discern the separation between the different encapsulation treatments and to identify the variables responsible for the separation. The score plots provide a visual representation of the sample distribution and the ability of the encapsulating agents to maintain the aroma profile of the jasmine instant tea. The OPLS-DA score plot for the jasmine instant tea, depicted in Figure S4 in the Supplementary Materials, demonstrates the variation in the samples’ aroma profiles post-encapsulation. Each sample is colored according to the encapsulating agent used, with the variation explained by the first predictive and orthogonal components (R2X [1] and R2X [2]). Similar to the PCA results, the plot reveals that the samples encapsulated with gelatin are more closely aligned with the fresh jasmine instant tea, suggesting that gelatin is more effective in preserving the aroma profile compared to acacia, CMC, and maltodextrin.

#### 2.5.3. Variable Importance in Projection (VIP) Analysis

The VIP scores from the OPLS-DA model provide insight into the importance of each variable in the model with respect to the projection used in the discriminant analysis. VIP scores greater than 1 suggest that a variable is important for the model, and scores greater than 1 are listed in Table 1 and Figure S5 in the Supplementary Materials, indicating that they were highly influential variables.

The impacts of different microcapsule wall materials on the retention and release of aroma compounds are closely related to the structural characteristics and types of these compounds. Esters, such as acetic acid phenylmethyl ester and methyl salicylate, are characterized by their -COOR functional groups, making them relatively non-polar and volatile. These esters are likely to interact more effectively with wall materials that have hydrophobic characteristics, such as gelatin, which possesses both hydrophilic and hydrophobic regions. This dual nature of gelatin allows it to form stable hydrogen bonds or hydrophobic interactions, leading to better retention of these volatile esters.

Siloxanes, including compounds like cyclotetrasiloxane and cycloheptasiloxane, are highly hydrophobic due to their silicon–oxygen backbone and attached methyl groups. Their encapsulation is more efficient in wall materials that are less polar, as hydrophobic interactions play a crucial role in their retention. Conversely, predominantly hydrophilic wall materials may lead to a greater loss of these volatile siloxanes during processing.

Alcohols, such as linalool and 3-hexen-1-ol, contain hydroxyl groups that make them somewhat polar, allowing them to form hydrogen bonds with wall materials like gelatin. This can enhance their retention, although their inherent volatility still poses a challenge, particularly during drying. Aromatic compounds, including indole and methyl anthranilate, contain benzene rings and other functional groups that contribute to their distinct odors. These compounds can interact with both hydrophilic and hydrophobic wall materials, and gelatin, with its ability to form complex interactions through hydrogen bonding and Van der Waals forces, can effectively retain these aromatic compounds.

Ketones, represented by compounds like 2-heptanone, have carbonyl groups that are moderately polar, allowing for potential hydrogen bonding with wall materials. However, due to their volatility, efficient encapsulation is necessary to minimize loss. The size and porosity of the microcapsules also play a significant role; larger microcapsules are better at encapsulating more material and reducing the release rate of volatile organic compounds. This is particularly important for highly volatile esters and siloxanes, which are prone to rapid evaporation. Overall, the choice of wall material is critical in determining the effectiveness of aroma compound retention, as it must provide a balance of hydrophilic and hydrophobic interactions, barrier properties, and encapsulation efficiency to preserve the desired aroma profile.

### 2.6. Heatmap Analysis

**Figure 5:**
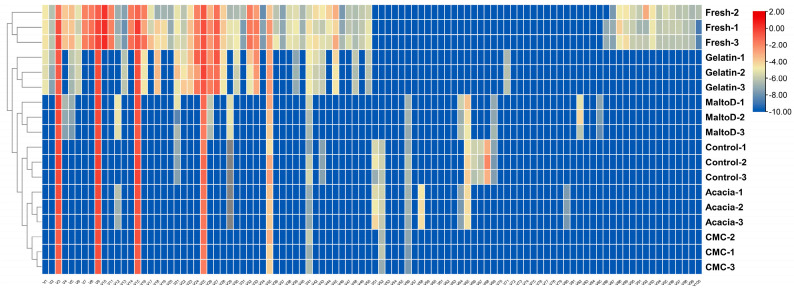
A heatmap of volatile compound expression levels in the microencapsulated tea samples. The heatmap displays the relative abundance of various volatile compounds across different microencapsulation samples and fresh tea. Color intensity reflects the expression levels, with higher concentrations shown in warmer colors and lower concentrations in cooler colors.

The heatmap in Figure 5 presents the expression levels of volatile organic compounds in microencapsulated jasmine instant tea, allowing for the comparison of profiles across different treatments and the control group. The color gradient on the heatmap, ranging from blue to red, indicates the relative intensity of the volatile organic compounds, with red representing higher expression levels and blue indicating lower levels. The dendrogram on the left side of the heatmap clusters the samples based on the similarity in their volatile organic compound profiles, providing insights into which samples have similar aroma profiles.

Each row in the heatmap corresponds to a different volatile organic compound, while each column represents a different sample. The clustering algorithm groups the samples based on the similarity in their volatile profiles, which are influenced by the encapsulating agents used in their preparation. The ‘Fresh’ samples are distinctly separated from the microencapsulated samples, indicating a divergence in the aroma profile post-encapsulation. Among the encapsulated samples, those treated with gelatin are clustered closer to the ‘Fresh’ samples, suggesting that gelatin encapsulation more effectively retains the original aroma profile.

The variability within the encapsulation groups, such as those seen in acacia and CMC, suggests that encapsulation with these agents results in a more heterogeneous retention of aroma compounds. conversely, the gelatin and maltodextrin samples demonstrate a more homogenous retention pattern, as reflected by the tighter clustering within these groups.

## 3. Conclusions

The findings presented in this study offer a comprehensive understanding of the role of microencapsulation in preserving aroma in jasmine instant tea. Through rigorous analysis, including PCA, OPLS-DA, permutation tests, and VIP scores, it is evident that the selection of encapsulating agents is crucial in maintaining the sensory qualities of jasmine instant tea. Notably, gelatin emerged as the most effective encapsulating agent, closely matching the volatile profile of fresh tea. This is particularly evident in the clustering patterns observed in the OPLS-DA plots, where gelatin-treated samples align more closely with fresh tea, indicating superior aroma preservation compared to other agents like acacia, CMC, and maltodextrin.

These results have important implications for the production of jasmine instant tea, suggesting that gelatin-based microencapsulation could bridge the gap between the convenience of instant tea and the sensory richness of traditionally brewed tea. For the tea industry, this could provide a significant competitive advantage in a market where sensory quality is a key determinant of consumer preference. Future research should focus on understanding the molecular interactions between gelatin and volatile organic compounds that result in this enhanced retention. Moreover, assessing the economic and environmental impacts of different encapsulating agents will be critical in ensuring that the selected methods are not only effective but also sustainable and cost-efficient. By building on these insights, the tea industry has the potential to develop products that are both high in sensory quality and aligned with sustainability and consumer health principles.

This research marks a significant advancement in addressing the challenge of aroma preservation in instant tea through advanced microencapsulation techniques. The optimization of encapsulation parameters has led to a notable improvement in the retention of volatile organic compounds, thereby enhancing the sensory profile of the final product. The use of novel homogenization techniques and comprehensive characterization of microcapsules using SEM and GC-MS has provided a deep understanding of the encapsulation process and its impact on instant tea quality. This study clearly demonstrates that microencapsulation can significantly reduce flavor loss during processing, offering a promising pathway for developing high-quality instant tea products. The scalability and applicability of the microencapsulation method developed here hold great potential for industrial adoption and could be extended to other food products where aroma is a critical quality attribute. Future work will concentrate on the long-term stability of encapsulated aromas under various storage conditions to further enhance the shelf life of instant tea. Additionally, sensory evaluations with larger consumer panels will provide deeper insights into consumer preferences and acceptance, further guiding product development.

## 4. Materials and Methods

### 4.1. Materials

Jasmine tea was purchased from Zhenjian Tea Co., Ltd. (Ya’an, China) and stored at room temperature (25 °C) in airtight containers to prevent moisture absorption and preserve its aroma before use. The encapsulating agents (gelatin (purity: ≥99%; CAS: 9000-70-8), acacia gum (purity: ≥98%; CAS: 9000-01-5)), carboxymethylcellulose (CMC) (purity: ≥99%; CAS: 9004-32-4), and maltodextrin (purity: ≥97%; CAS: 9050-36-6),) were provided by Macklin Biochemical Technology Co., Ltd. (Shanghai, China). All the encapsulating agents were stored in a cool, dry place (15–25 °C) until use. The internal standard ethyl decanoate (purity: ≥98%; CAS: 110-38-3) was purchased from Shanghai Aladdin Biochemical Technology Co., Ltd. (Shanghai, China) and stored at room temperature. Other chemical reagents, including ethanol (purity: ≥99.8%; CAS: 64-17-5), Folin phenol reagent (purity: 99%; CAS: 12111-13-6), sodium carbonate (Na_2_CO_3_) (purity: ≥99.5%; CAS: 497-19-8), and vitamin C (Vc) (purity: ≥99%; CAS: 50-81-7), were purchased from the market.

### 4.2. Encapsulation of Jasmine Tea

Jasmine tea leaves were ground into a fine powder and passed through a 100-mesh sieve to ensure a uniform particle size. A total of 25 g of the jasmine tea powder was steeped in 1250 mL of boiling distilled water for 30 min to conduct the first extraction. Afterward, the tea residue was collected, and a second extraction was carried out by steeping the residue in an additional 1250 mL of boiling distilled water for another 30 min. The filtrates from both extractions were combined, yielding a total of 2500 mL of jasmine tea extract. A total of 2.5 g of each encapsulating agent (gelatin, acacia gum, carboxymethylcellulose (CMC), or maltodextrin) was added to the tea extract. The mixtures were homogenized using a high-speed homogenizer (FSH-2A, Jintan Instrument Co., Ltd., Changzhou, China) at a speed of 10,000 rpm for 5 min to ensure uniform dispersion of the encapsulating agents. The tea infusion, with or without the encapsulating agents, was concentrated using a rotary evaporator (Buchi R-300, Büchi Labortechnik AG, Flawil, Switzerland) at 55 °C under reduced pressure (0.1 MPa) until the volume was reduced to 250 mL. This concentrated tea infusion was then subjected to spray drying using a spray dryer (ADL311S, Yamato Scientific Co., Ltd., Tokyo, Japan) with an inlet temperature of 160 °C and an outlet temperature of 60 °C, resulting in the production of jasmine instant tea powder.

Encapsulation efficiency (EE) was evaluated by measuring the total phenolic contents in the sample prior to encapsulation (TPtotal) and in the supernatant after centrifugation (TPsupernatant). The EE (%) was calculated with the following formula:

EE (%) = (TPtotal − TPsupernatant)/TPtotal × 100% (1)

### 4.3. Particle Size

The particle size of the sample was measured by a laser particle sizer (Mastersizer 3000, Malvern Panalytical Ltd., Malvern, UK). Prior to measurement, the samples were diluted tenfold with ultrapure water. All measurements were conducted at 25 °C and repeated three times.

### 4.4. SEM

The morphological features of the jasmine instant tea powders were analyzed using Scanning Electron Microscopy (SEM). The samples were secured onto conductive supports using conductive glue. They were then coated with gold–palladium and examined with a Phenom Pro SEM (Phenom World, Eindhoven, Eindhoven, The Netherlands), with the acceleration voltage set at 10 kV. The SEM images reveal distinct surface characteristics of the microcapsules, including surface smoothness, the presence of pores, and occasional cracks. The surface morphology plays a critical role in influencing the physical properties of instant tea powders. Microcapsules with smoother surfaces exhibit fewer cracks and pores, which are essential for enhancing their protective capabilities. These surface features play a significant role in the encapsulation efficiency and stability of the volatile organic compounds, directly impacting the overall quality of the instant tea.

### 4.5. Polyphenols and Flavonoids

For the extraction of samples used to determine the encapsulation efficiency, polyphenols and flavonoids from the jasmine instant tea microcapsules were directly extracted using 95% ethanol. For the samples used to measure the total phenolic and flavonoid content, an ultrasonic cell disruptor was used to break the samples for 10 min, followed by extraction with 95% ethanol.

Folinphenol colorimetry was used to analyze the content of polyphenols in the tea extract [40]. A sample volume of 0.5 mL of Folin phenol was taken to start the reaction for 3 min, and 1 mL of 15% sodium carbonate Na_2_CO_3_ solution was added to the mixture solution and left to stand for 30 min. Then, the reaction mixture was centrifuged at 3500 RPM for 3 min to separate the phases, and the supernatant was carefully collected for analysis. The absorbance of the supernatant was measured at 760 nm using a spectrophotometer. The spectrophotometer was zeroed with a 15% Na_2_CO_3_ solution to ensure accurate measurements. Catechins were used to plot the standard curve, and the total phenolic content results were expressed in catechin equivalents.

### 4.6. Antioxidant Capacity

The DPPH (2,2-diphenyl-1-picrylhydrazyl) assay is an effective method to evaluate the free radical scavenging activity of antioxidants [40]. The standard curve, plotted with absorbance at 517 nm, helped in quantifying the antioxidant capacity of the samples. The absorbance values obtained for the Vc standard varied from 1.593 to 1.195, corresponding to Vc concentrations ranging from 0 to 10 µL. The regression equation obtained from the standard curve was y = −0.0509 + 1.6451, with an R2 = 0.99.

### 4.7. SPME-GC-MS Analysis of Aroma Compounds

Solid-Phase Microextraction Gas Chromatography–Mass Spectrometry (SPME-GC-MS) [40] was employed to determine the volatile organic compounds in jasmine instant tea. The analysis utilized a CHIRAMIX column to achieve high precision.

For powdered samples, 1 g was dissolved in 25 mL of boiling water, followed by the addition of 5 μL of the internal standard ethyl decanoate. A 5 mL portion of this mixture was transferred into a 20 mL sealed headspace vial. This procedure was repeated three times for each sample to ensure reproducibility. For liquid samples obtained post-concentration, 0.5 μL of the internal standard was thoroughly mixed in, and 5 mL of this blend was placed in a 20 mL sealed headspace vial for analysis.

The headspace SPME fiber, coated with 65 μm polydimethylsiloxane/divinylbenzene (PDMS/DVB), was exposed to the sample headspace for 30 min at 80 °C to efficiently absorb volatile organic compounds. After extraction, the fiber was inserted into a GC-MS spitless injector at 250 °C for 3.5 min for desorption. The separation and identification of the compounds were carried out using helium as the carrier gas at a flow rate of 1 mL/min. The temperature program began at 50 °C, increased to 210 °C at a rate of 3 °C/min (and held for 3 min), and then from 210 °C to 230 °C at 14 °C/min. The mass spectrometer’s ion source temperature was set at 230 °C with an electron energy of 70 eV, scanning over a range of 30–500 amu, and employing a solvent delay time of 2.8 min.

### 4.8. Statistical Analysis

Statistical analyses were performed in SPSS 22.0 (SPSS Inc., Chicago, IL, USA) using an analysis of variance (ANOVA) and Tukey multiple comparison, and repeated three times. The PCA and OPLS-DA analyses were conducted with Simca 14.1 (Malmö, Sweden). All results are expressed as means ± standard deviations of the replications (*p* < 0.05).

## Figures and Tables

**Table 1:** The aroma compounds with VIP values higher than 1.

Var ID	Aroma Compound	VIP Value
V10	Acetic acid, phenylmethyl ester	2.93
V3	Cyclotetrasiloxane, octamethyl-	2.37
V27	Undecanoic acid, ethyl ester	2.18
V68	Butylated hydroxytoluene	2.08
V25	Cycloheptasiloxane, tetradecamethyl-	1.89
V58	Cyclotrisiloxane, hexamethyl-	1.81
V24	2(3H)-Furanone, dihydro-5-propyl-	1.78
V9	Cyclopentasiloxane, decamethyl-	1.61
V15	Cyclohexasiloxane, dodecamethyl-	1.60
V8	Linalool	1.58
V51	Oxime-, methoxy-phenyl-_	1.56
V26	2H-Pyran, 2-[(5-cyclopropylidenepentyl)oxy]tetrahydro-	1.56
V14	Indole	1.49
V32	3-Hexen-1-ol, benzoate, (Z)-	1.47
V11	Methyl salicylate	1.42
V7	Benzoic acid, methyl ester	1.40
V33	Dodecanoic acid, ethyl ester	1.38
V16	Methyl anthranilate	1.35
V82	2(3H)-Furanone, 5-hexyldihydro-	1.25
V65	2-Heptanone, 5-methyl-	1.22
V12	Decanal	1.18

## Data Availability

The original contributions presented in the study are included in the article/Supplementary Material, further inquiries can be directed to the corresponding author/s.

## Supplementary Materials

The following supporting information can be downloaded at: https://www.mdpi.com/article/10.3390/gels10100670/s1, Figure S1. Particle size distribution of microencapsulations produced using various wall materials. This figure illustrates the size range of microencapsulations fabricated with different wall materials, including gelatin, acacia gum, carboxymethylcellulose (CMC), and maltodextrin; Figure S2. Chromatogram of tea infusion by the tea sample using various wall materials. A: Fresh tea; B: control group (without microencapsulation); C: acacia gum; D: carboxymethylcellulose (CMC); E: gelatin; and F: maltodextrin; Figure S3. Permutation test results from the Orthogonal Partial Least Squares Discriminant Analysis (OPLS-DA) model. This figure shows the permuted values for the OPLS-DA model, indicating the significance and robustness of the model in distinguishing between different microencapsulations. Each bar represents a permutation, with the observed value plotted against the permuted distribution to assess model reliability; Figure S4. Orthogonal Partial Least Squares Discriminant Analysis (OPLS-DA) plot comparing different microencapsulation samples with fresh tea samples. The plot illustrates the separation and clustering of microencapsulations made from various wall materials (gelatin, acacia gum, carboxymethylcellulose (CMC), maltodextrin) relative to fresh tea, based on their aroma profiles; Figure S5. Variable Importance in Projection (VIP) scores from the Orthogonal Partial Least Squares Discriminant Analysis (OPLS-DA) model for jasmine instant tea. This figure displays the VIP scores for different variables, indicating their significance in differentiating between various jasmine instant tea samples. Higher VIP scores denote greater importance of the variables in the model’s predictive capability; Table S1. The volatiles organic compounds analyzed by SPME-GC-MS.
