# Supplementary material for: Microencapsulation Efficiency of Carboxymethylcellulose, Gelatin, Maltodextrin, and Acacia for Aroma Preservation in Jasmine Instant Tea
Source: Gels. 2024 Oct 21;10(10):670. doi: 10.3390/gels10100670 (PMC11507381; doi:10.3390/gels10100670)
Supplement: Supplementary file 1 [file gels-10-00670-s001.zip › gels-3220719-supplementary.pdf]

Supplementary File

Microencapsulation Efficiency of Carboxymethylcellulose, Gelatin, Maltodextrin, and Acacia for Aroma Preservation in Jasmine Instant Tea

Muneeba Naseer Chaudhary [1], Xiaolin Li [1], Siyue Yang [1], Damao Wang [2], Liyong Luo [1,3], Liang Zeng [1,3,] * and Wei Luo [1,3,] *

[1] Integrative Science Center of Germplasm Creation in Western China (CHONGQING) Science City /College of Food Science, Southwest University, Chongqing 400715, PR China
[2] College of Food Science, Southwest University, Chongqing 400715, PR China
[3] Chongqing Key Laboratory of Speciality Food Co-Built by Sichuan and Chongqing, Southwest University, Chongqing 400715, PR China
* Correspondence: Wei Luo (luowei1900@swu.edu.cn; address: College of Food Science, Southwest University, Chongqing 400715, PR China)
* Correspondence: Liang Zeng (E-mail: zengliangbaby@126.com; address: College of Food Science, Southwest University, Chongqing 400715, PR China)

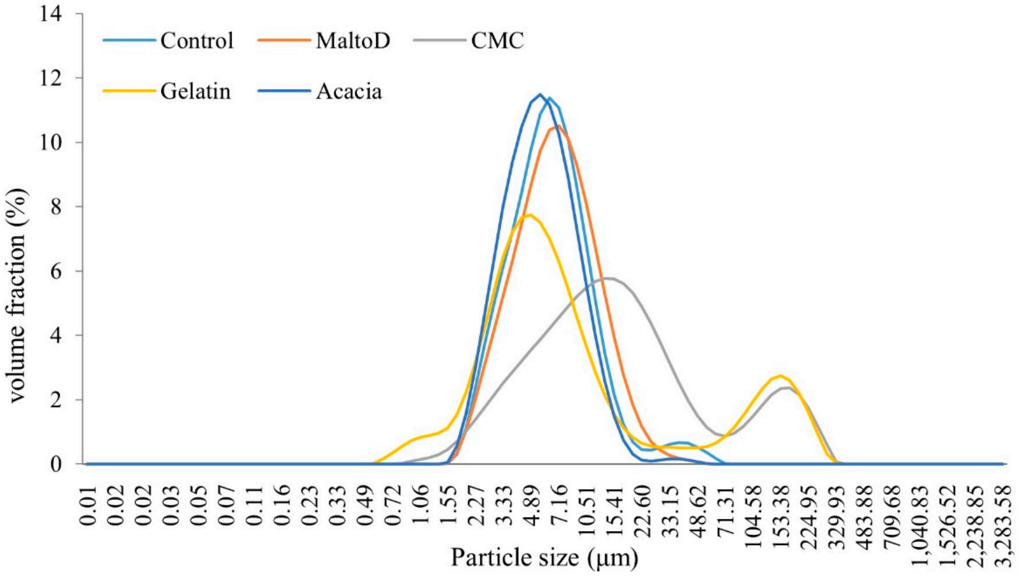

Figure S1. Particle size distribution of microencapsulations produced using various wall materials. This figure illustrates the size range of microencapsulations fabricated with different wall materials, including gelatin, acacia gum, carboxymethylcellulose (CMC), and maltodextrin.

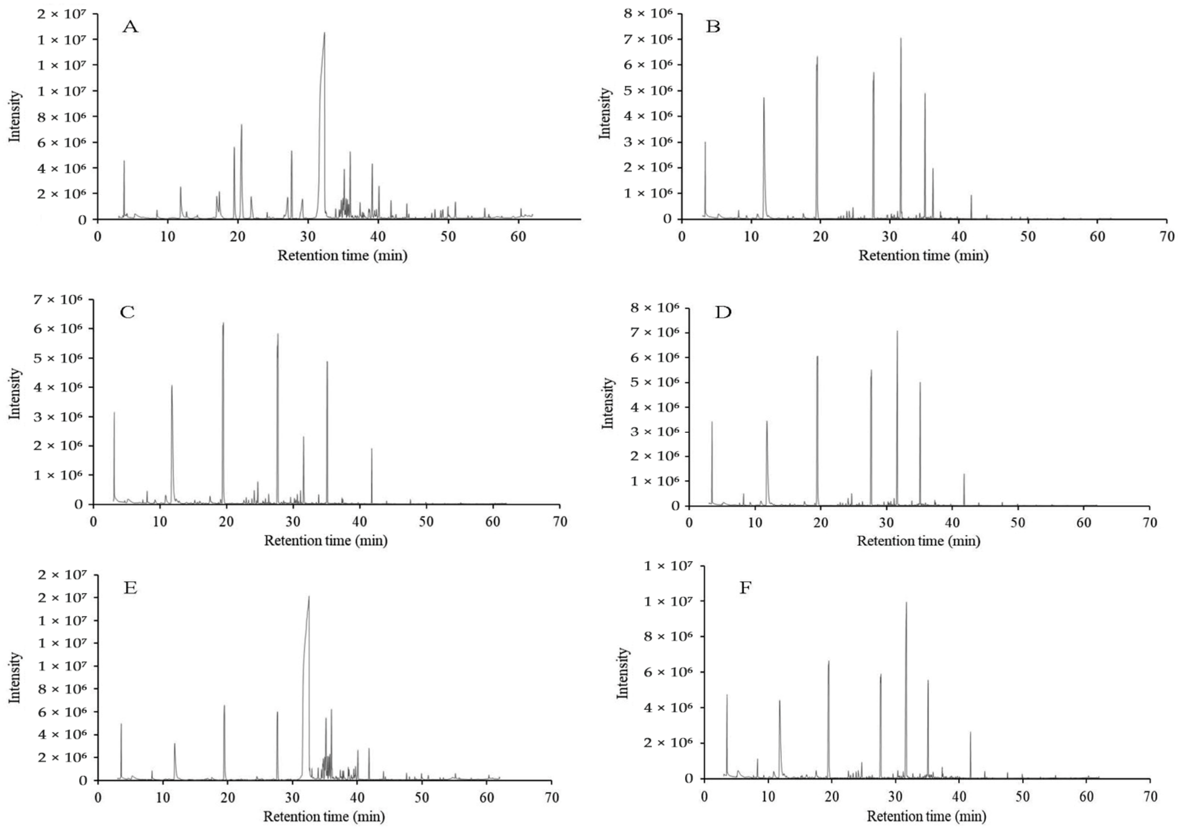

Figure S2. Chromatogram of tea infusion by the tea sample using various wall materials. A: Fresh tea; B: control group (without microencapsulation); C: acacia gum; D: carboxymethylcellulose (CMC); E: gelatin; and F: maltodextrin.

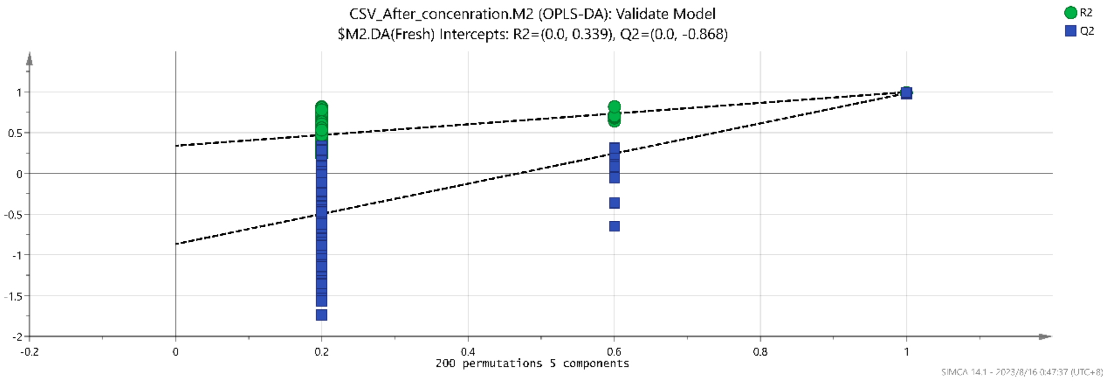

Figure S3. Permutation test results from the Orthogonal Partial Least Squares Discriminant Analysis (OPLS-DA) model. This figure shows the permuted values for the OPLS-DA model, indicating the significance and robustness of the model in distinguishing between different microencapsulations. Each bar represents a permutation, with the observed value plotted against the permuted distribution to assess model reliability.

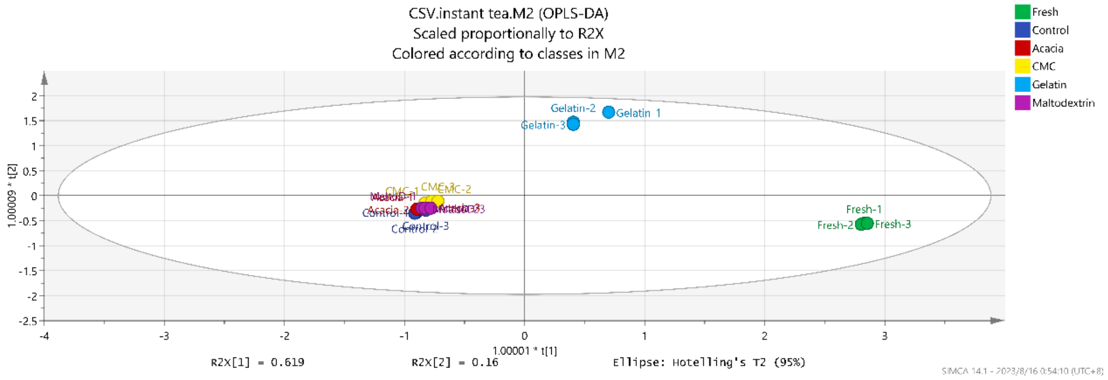

Figure S4. Orthogonal Partial Least Squares Discriminant Analysis (OPLS-DA) plot comparing different microencapsulation samples with fresh tea samples. The plot illustrates the separation and clustering of microencapsulations made from various wall materials (gelatin, acacia gum, carboxymethylcellulose (CMC), maltodextrin) relative to fresh tea, based on their aroma profiles.

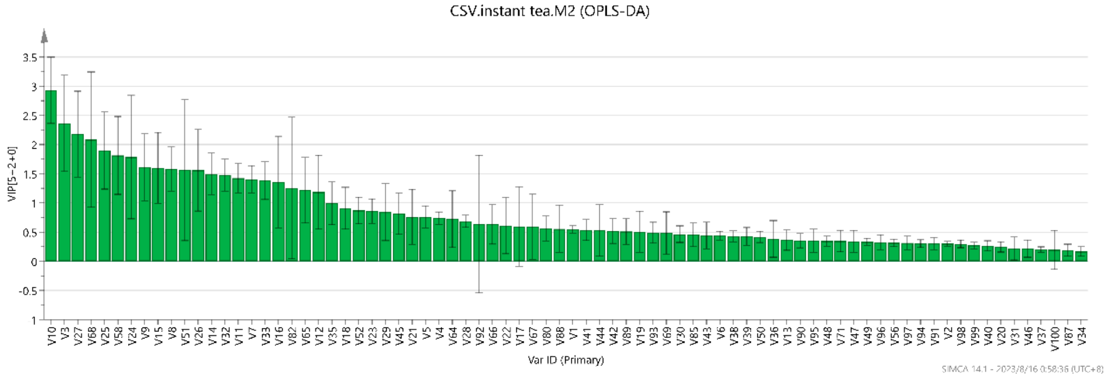

Figure S5. Variable Importance in Projection (VIP) scores from the Orthogonal Partial Least Squares Discriminant Analysis (OPLS-DA) model for jasmine instant tea. This figure displays the VIP scores for different variables, indicating their significance in differentiating between various jasmine instant tea samples. Higher VIP scores denote greater importance of the variables in the model's predictive capability.

Table S1. The volatiles organic compounds analyzed by SPME-GC-MS.

	2H-Pyrann	5-Hepten-	Cyclotetras	3-Hexen-1	Benzyl alcc	Hexanoic a
	V1	V2	V3	V4	V5	V6
Fresh-1	0.038237	0.012257	0.702194	0.071552	0.071257	0.017455
Fresh-3	0.036736	0.011912	0.355976	0.069088	0.083289	0.028029
Fresh-3	0.041751	0.013355	0.720566	0.078831	0.101218	0.027431
Control-1	0	0	0.777234	0	0	0
Control-2	0	0	0.935146	0	0	0
Control-3	0	0	0.769745	0	0	0
Acacia-1	0	0	0.728937	0	0	0
Acacia-2	0	0	0.714522	0	0	0
